# Comparison of Tranexamic Acid and Dexmedetomidine on Bleeding in Endoscopic Sinus Surgery

**DOI:** 10.22038/IJORL.2022.64361.3203

**Published:** 2023-01

**Authors:** Mohammad Saeed Ahmadi, Javaneh Jahanshahi, Farnaz Hashemian, Ahmad Reza Salimbahrani, Negar Haghi, Elham Khanlarzadeh

**Affiliations:** 1 *Department of Otolaryngology, Hamadan University of Medical Sciences, Hamadan, Iran.*; 2 *Department of Anesthesiology, Hamadan University of Medical Sciences, Hamadan, Iran.*; 3 *Department of Social Medicine, Hamadan University of Medical Sciences, Hamadan, Iran.*

**Keywords:** Chronic rhinosinusitis, Dexmedetomidine, Tranexamic acid, Sinus endoscopy

## Abstract

**Introduction:**

The quality of the surgical field during the surgery is impaired when bleeding occurs. This study compared the effect of tranexamic acid and dexmedetomidine on the rate of bleeding during endoscopic sinus surgery (ESS).

**Materials and Methods:**

In this one-blind clinical trial, 72 patients with chronic rhinosinusitis who were candidates for ESS at Be’sat Hospital in Hamedan were randomly assigned to two groups. Group A received dexmedetomidine at a dose of 1μg/kg, and group B received tranexamic acid at a dose of 10mg/kg immediately after induction of anesthesia intravenously within 15 minutes. The two groups were evaluated and compared regarding the quality of the surgery field with the Boezaart scale, volume of intraoperative bleeding, hemodynamic changes, and complications up to 90 minutes after the beginning of surgery.

**Results:**

The mean volume of intraoperative bleeding in group A (181.67±86.66) was significantly higher than in group B (110.28±61.23) (P =0.000). At 15, 30, and 60 minutes, the quality of the surgical field in group B was better than group A (P =0.038), while at 90 minutes, there was no statistically significant difference (P =0.450). The mean arterial pressure in group A at 15 minutes was higher than in group B (P=0.003); at 60 and 90 minutes, it was lower, and the difference was statistically significant (P =0.01). On the other hand, in 30 minutes, the mean arterial pressure in group A was higher than in group B, without a significant difference (P =0.07). Moreover, there was no statistically significant difference between the average surgery time (P = 0.25) and the frequency of complications (P =0.405).

**Conclusions:**

Based on the results, tranexamic acid is preferable to injectable dexmedetomidine to control and reduce bleeding during ESS.

## Introduction

Chronic sinusitis is one of the most common chronic diseases that affect people of all ages (1). Rhinosinusitis is an inflammation of the paranasal sinuses, which may be caused by an infection, allergy, or autoimmune disease (2). Treatment of chronic rhinosinusitis is based on three principles: controlling or stopping the growth of bacteria, reducing mucosal swelling, and lubricating fluid within the sinuses (3,4). Endoscopic surgery is one of the chronic rhinosinusitis’s most common surgical procedures (5). Bleeding during endoscopic sinus surgery is one of the most common problems in this operation, so it is difficult for the surgeon to establish a favorable surgical field, and even a small hemorrhage may distort the endoscopic vision, leading to longer surgery and even incomplete surgery or rarely causes complications such as diplopia, blindness, CSF leak, damage to the internal carotid artery. In order to improve the field of sinus surgery, many methods have been suggested, including bipolar diathermy, packing, local vasoconstrictors, and induced hypotension. Reducing bleeding by an anesthesiologist is very important to increase the surgeon's vision. Controlled blood pressure lowering to reduce bleeding is valuable in improving the surgeon's vision during sinus endoscopy (5,6).

Various techniques can reduce bleeding, which can be done by hemodynamic methods such as controlled blood pressure lowering, local vasoconstrictors, epidural block, biological and chemical drugs such as desmopressin, aprotinin (7), tranexamic acid (8, 9), epsilon aminocaproic acid, and estrogen. However, neither is ideal on its own. The ideal way to control bleeding depends on several factors, such as how it is administered, the onset of action, the time required to return, and excretion without producing toxic metabolites in the event of a problem (5,6,10).

Dexmedetomidine, as an alpha-dual adrenergic receptor agonist, is 1620 times more potent than the alpha-1 adrenergic receptor, and in healthy individuals, on average, 94% of the drug is bound to serum albumin and alpha-1 glycoprotein (11). By acting on these receptors, it is thought that this drug leads to the activation of G proteins and inhibition of norepinephrine secretion, followed by hypotension. The hypnotic, analgesic and hypotensive effects are dose-dependent. The drug’s effect is 5 to 10 minutes after the start of the injection, the peak effect is 15 to 30 minutes, and the half-life is between one to two hours. The drug is currently available under Presidex and Sedodex in one and two milliliters vials containing 100μg/cc. This drug is a new sedative and analgesic with important benefits, such as rapid effectiveness and titration, changing the depth of sedation and analgesia, and a synergistic effect with existing anesthetics. It also has a low rate of drug side effects and respiratory depression (12).

Tranexamic acid (TA) is a synthetic antifibrinolytic substance (lysine analog) that binds to plasmin and plasminogen (13) and inhibits their ability to bind to lysine residues in fibrin and therefore prevents fibrinolysis (13, 14). Any surgery can release enzymes, such as tissue plasminogen activators, with significant tissue damage; the mechanism of action of TA is that by inhibiting the activity of this enzyme, it can prevent fibrinolysis activity (15).

The present study was performed to compare the effect of dexmedetomidine and tranexamic acid on bleeding rate and improve field quality during endoscopic sinus surgery in patients with rhinosinusitis (with and without polyposis) and sinus endoscopy candidate referred to Be’sat Hospital in Hamadan.

## Materials and Methods

This randomized one-blind clinical trial study was conducted on 72 patients with chronic rhinosinusitis who were candidates for endoscopic surgery (with and without polyposis). Patients referred to Be’sat Hospital in Hamadan in 2021 were admitted based on inclusion and exclusion criteria (with the ethical committee code IR.UMSHA.REC.1399.298). One-blind clinical trial study means that the drugs A and B were drawn in syringes of the same color and shape by the anesthesia technician, and the anesthesiologist at the patient's bedside was aware of the type of injected drug. Two senior surgeons performed the surgery, and none of the surgeons were aware of the type of medicine.

Inclusion criteria were patient satisfaction, with age between 18 and 60 years and no history of bleeding disorders, thromboembolic events, liver and kidney failure, uncontrolled systemic diseases such as hypertension, diabetes, heart failure, and arrhythmias, cardiac stents, use of anti-inflammatory drugs Coagulants for up to five days prior to surgery. Pregnant women and those with a history of allergy to these two drugs were excluded from the study. The following formula was used for sample size calculation based on comparing the average between the two groups:

$n=\frac{{\left(Z_{1-\frac{\alpha }{2}}+Z_{1-\beta }\right)}^{2}\times (S_{1}^{2}+S_{2}^{2})}{{\left(\mu_{1}-\mu_{2}\right)}^{2}}$

Concerning α=0.05 and β=0.2, and based on a study conducted by Baranjian et al. (20), µ_1_ and µ_2_ equal to 1.42 and 1.65, respectively, and s_1_ and s_2_ equal to 0.53 and 0.64, respectively (the mean and standard deviation of the quality of the surgical field in two groups of tranexamic acid and dexmedetomidine), the sample size for each group was estimated to be 36 people.

Patients were randomly divided into tranexamic acid and dexmedetomidine groups of 36 using a randomized six-block method by a person who knew the type of drug encoded in each group. For both groups, preoperative PNS CT SCAN axial & coronal CT scans were performed, and the severity of sinus was scored according to Lund Mackey criteria. CBC and coagulation tests (PT, PTT, INR, BT, and CT) were among the tasks that were performed one day before surgery. Before surgery, an intravenous route was established for all patients, and routine operating room monitoring included non-invasive blood pressure measurement, ECG monitoring, and pulse oximetry. Immediately after induction of anesthesia, tranexamic acid at a dose of 10mg/kg was intravenously injected into the first group for over 15 minutes. Dexmedetomidine was similarly injected in the second group at a dose of 1μg/kg. Two senior surgeons performed the surgery, and neither was aware of the type of medication. The anesthesiologist at the patient's bedside was aware of the type of injectable drug.

Anesthesia was performed using 5-7g/kg fentanyl, 1-2 mg/kg propofol, 0.1mg/kg midazolam, and 0.5mg/kg atracurium relaxant to induction of anesthesia and intubation. Infusion of fentanyl at 1-3g/kg/h and propofol at 100-200µg/kg/min was performed to maintain moderate arterial blood pressure in the range of 5 to 7 mmHg and anesthesia management. Moderate arterial blood pressure was recorded before and after surgery. The surgery was performed by a single surgical team. The quality of vision of the surgical field at 15, 30, 60, and 90 minutes after the start of surgery based on Boezaart Grading and also the amount of bleeding during the operation at 15, 30, 60, and 90 minutes according to the amount of blood collected in the suction bottle (after decreasing from the volume of the used normal saline). At the same time, mean arterial blood pressure was also recorded. According to the patient's weight and blood loss, normal saline is prescribed during surgery. All information about age, sex, and other demographic factors were collected and recorded on a special sheet. Bleeding rate, blood pressure, quality of the surgical field, and duration of surgery were recorded in the data collection form.

The Mann-Whitney test and t-test were used to compare the means of bleeding amount during endoscopic surgery (FESS), surgical field quality score (Boezaart score), hemodynamic changes, and time between the two groups. Complications were compared by the Chi-square test. Moreover, a comparison of the mean bleeding amount during endoscopic surgery and the quality score of the surgical field according to the severity of sinus involvement was performed using repeated measures. The most important assessment items in determining drug side effects included bradycardia, blood pressure, nausea, vomiting, tachycardia, dry mouth, and high blood pressure. A P-value less than 5% was considered statistically significant.

*Findings*

This study considered 72 patients (with and without polyposis) in two equal groups of 36 tranexamic acid and dexmedetomidine. In the tranexamic acid group, with a mean age of 37.63±9.95 patients, 19 were male (52.8%), and 17 were female (47.2%). 

In the dexmedeto- midine group, the mean age of the patients was 34.55±10.78, of which 21 were male (58.3%), and 15 were female (41.7%). There was no significant difference between the two groups in gender and age (P.v>0.05) (Table 1).

**Table 1:** Demographic information of patients in two therapy groups

**P.value**	**Group of therapy**		**Variable**
	**Dexmedetomidine**	**Tranexamic**	
0.21	34.55±10.78	37.63±9.95	Age (years)
0.6	21(58.3%)	19(52.8%)	Sex (male)
0.6	15(41.7%)	17(47.2%)	Sex (Female)

The severity of sinus involvement was evaluated based on the PNS CT scan performed for all patients before surgery and the determination of CT score based on Lund Mackey criteria. According to Table 2, there was no significant difference in the severity of sinus involvement in the two groups of patients undergoing endoscopic sinus surgery.

**Table 2:** Comparison of the severity of sinus involvement based on CT score (Lund_Mackey) in patients of the two treatment groups

**P.value**	**Group of therapy**		**The severity of sinus involvement** **(Based on CT scan score)**
	**Dexmedetomidine**	**Tranexamic**	
0.06	9.2222	8.2222	Lund_Mackey. Right. Total
0.36	8.6667	8.0556	Lund_Mackey. Left. Total
0.56	17.7889	16.2778	Lund_Mackey. Score. Total

In both groups, none of the patients had a preoperative bleeding disorder and an INR greater than 1.5. The amount of bleeding during the operation was calculated in two groups. Based on the results of the Mann-Whitney test and t-test, there was a significant difference between tranexamic acid and dexmedetomidine 

groups in terms of intraoperative bleeding volume at the measured times of 15, 30, 60, and 90 minutes. At all times, the rate of bleeding was significantly higher in the dexmedeto- midine group. However, in these periods, it still had fewer effects in reducing bleeding volume than tranexamic acid (Table 3).

**Table 3:** Comparison of mean bleeding volume in milliliters during FESS between the two treatment groups

**P.value**	**Group of therapy**		**Time**
	**Dexmedetomidine**	**Tranexamic**	
0.002	71.38±45.28	37.22±30.29	15 minutes
0.000	120.28±50.26	61.38±46.43	30 minutes
0.000	153.89±61.88	90.55±51.43	60 minutes
0.000	181.67±86.66	110.28±61.23	90 minutes

Due to the non-normal distribution of the Boezaart Grading variable and Mann-Whitney test, the quality of the surgical field in the tranexamic acid group was better than the dexmedetomidine group. The difference was significant at 15, 30, and 45 minutes; but not at 90 minutes (Table 4).

**Table 4:** Comparison of the average quality score of the surgical field based on the Boezaart Grading

**P.value**	**Group of therapy**		**Time**
	**Dexmedetomidine**	**Tranexamic**	
0.004	2.33±0.95	1.72±0.88	15 minutes
0.000	2.77±0.86	1.77±0.86	30 minutes
0.038	2.33±0.95	1.88±0.86	60 minutes
0.450	2.08±1.05	1.88±0.94	90 minutes

As seen in Table (5), there was a direct and significant relationship between the severity of sinus involvement based on CT SCORE and the bleeding volume at all times. The more intense the conflict, the more bleeding. 

In addition, there was a direct and significant relationship between the severity of sinus involvement and the quality score of the surgical field.

**Table 5:** Considering the severity of sinus involvement correlation with the volume of intraoperative bleeding and quality of the surgical field for the two treatment groups

**Spearman correlation coefficient R, correlation of sinus involvement severity: Lund_Mackey. Score. ** **Total with:**				
P.value	Quality of surgical field (Boezaart)	P.value	Bleeding volume	Time
0.017	0.25	0.003	0.316	15 minutes
0.03	0.21	0.001	0.367	30 minutes
0.01	0.26	0.002	0.335	60 minutes
0.06	0.18	0.002	0.340	90 minutes

Analysis of data to compare changes in mean arterial pressure (MAP) in patients in tranexamic acid and dexmedetomidine groups at different times of the evaluation suggested a significant difference, so at 15 minutes, the MAP in the dexmedetomidine group was higher than tranexamic acid and less in the 60 and 90 minutes. Besides, the MAP at 15 minutes in the dexmedetomidine group was higher than the tranexamic acid group, but the difference was not statistically significant (Table 6).

**Table 6:** Comparison of MAP variations between the two treatment groups

**P.value**	**Group of therapy**		
	**Dexmedetomidine**	**Tranexamic**	
**0.08**	**108.74±10.96**	**104.19±11.79**	**Before surgery**
0.003	97.29±13.13	85.92±16.67	15 minutes
0.07	83.03±12.52	79.29±14.50	30 minutes
0.003	69.81±10.75	77.46±14.16	60 minutes
0.01	67.59±8.54	76.16±13.72	90 minutes

The analysis of complications in the two groups showed that seven patients (19.4%) in the tranexamic acid group and ten patients (27.8%) in dexmedetomidine had complications. None of the complications were major or life-threatening. The chi-square test showed no significant difference in the complications between the two study groups (P.v= 0.29) (Table 7).

**Table 7:** Frequency distribution of complications in two treatment groups

**P.value**	**Group of therapy**		**Complications**
	**Dexmedetomidine**	**Tranexamic**	
0.001	4(11.1%)	0(0%)	Bradycardia
	4(11.1%)	0(0%)	Hypertension
	0(0%)	3(8.3%)	Nausea
	0(0%)	3(8.3%)	Vomit
	1(2.8%)	0(0%)	Tachycardia
	0(0%)	1(2.8%)	Dry mouth
	1(2.8%)	0(0%)	Hypotension
0.405	10(27.8%)	7(19.4%)	Total

The duration of surgery was 140.61±57.03 minutes in the tranexamic acid group and 153.56±35.74 minutes in the dexmedetomidine group. In the independent t-test, the surgery duration in the dexmedetomidine group was longer than tranexamic acid, but the difference was not statistically significant (P.v= 0.25).

## Discussion

Bleeding is one of the main considerations in sinus surgeries; the field operated on is very vascular and bloody. Bleeding can be venous, capillary, or arterial, leading to serious complications due to poor vision during surgery. Creating ideal homeostasis with antifibrinolytics in modern surgery is one of the treatment principles. Various studies have shown the effectiveness of tranexamic acid in injured patients in reducing bleeding and frequent blood transfusions (16). The drug tranexamic acid blocks fibrinolysis by blocking lysine on plasminogen and prevents bleeding. Intraoperative tranexamic acid administration has been performed in different populations of injured and non-injured patients undergoing different surgeries. In many surgeries, tranexamic acid is still a very effective antifibrinolytic drug. Studies on the anti-hemorrhagic effects of this drug in ENT surgeries also show significant benefits of this drug (17,18).

However, it is notable that the amount of bleeding in the dexmedetomidine group in 30 to 90 minutes was less severe than the bleeding in the first 30 minutes, which can be justified by the mechanism and how dexmedetomidine works. Dexmedetomidine, an alpha-dual adrenergic receptor agonist, inhibits norepinephrine secretion, leading to hypotension. The drug effect is 5 to 10 minutes after the start of the injection, the peak effect is 15 to 30 minutes, and the half-life is between one to two hours. Therefore, after the drug reaches its peak, it significantly controls the bleeding volume compared to the first 30 minutes of surgery. 

In a clinical trial conducted by Modiri et al. (2021), 105 patients were randomly divided into three groups for septorhinoplasty. This trial was conducted on the efficacy and safety of tranexamic acid, dexmedetomidine, and nitroglycerin in controlling intraoperative bleeding and improving the quality of the surgical field during septorhinoplasty under general anesthesia. The first group received 1μg/kg dexmedetomidine, the second group received 10mg/kg tranexamic acid, and the third group received 0.5μg/kg nitroglycerin intravenously. The results showed no statistically significant difference between the three groups regarding bleeding rate and surgeon satisfaction with the surgical field (19).

In the present study, tranexamic acid significantly reduced the bleeding volume at all times compared to the dexmedetomidine group. Tranexamic acid can reduce bleeding in the operating field and increase its surgical quality by creating a relatively dry and bleeding-free field. The difference was statistically significant at 15, 30, and 60 but not at 90 minutes. The better efficacy of tranexamic acid may be related to the mechanism of the drug. By binding to plasmin and plasminogen, tranexamic acid inhibits their ability to bind to lysine residues in fibrin and thus prevents fibrinolysis, resulting in reduced bleeding and improving the quality of the surgical field.

In 2017, Hassani et al. compared the effect of tranexamic acid and dexmedetomidine on bleeding during rhinoplasty during a double-blind clinical trial. In this randomized study, 76 patients were candidates for elective rhinoplasty. Tranexamic acid, at a dose of 10mg/kg, was intravenously administrated within 15 minutes immediately after the induction of anesthesia in the first group. In the second group, dexmedetomidine was similarly injected at a dose of 1μg/kg. The rate of intraoperative bleeding in the dexmedetomidine and tranexamic acid groups was 145.2±22.1 and 141.8±24.1 ml, and the mean score of the surgeon's satisfaction was 1.65±0.53 and 1.42±0.64, respectively. In total, the efficacy of dexmedetomidine and tranexamic acid was equal. The average drop in arterial pressure in the dexmedetomidine group was much lower than in the tranexamic acid group (20).

In a double-blind clinical trial performed by Mathew et al. in 2019 on intravenous dexmedetomidine injection in hemodynamic control of surgical field and recovery profile, 40 patients 18 to 65 years old of ASA I-II class with transsphenoidal pituitary surgery were divided into two groups of dexmedetomidine 1μg/kg and placebo. The results showed no significant difference between the two groups regarding baseline heart rate during surgery and recovery. Mean systolic and diastolic blood pressure decreased significantly in the dexmedetomidine group. However, it was within the clinically acceptable range and did not require intervention. Bleeding scores during surgery and recovery were similar between the two groups (21).

In the present study, similar to the last two studies, the mean arterial pressure at 60 and 90 minutes was lower in the dexmedetomidine group than in the tranexamic acid group. Dexmedetomidine, as an alpha-2 receptor agonist, inhibits the secretion of norepinephrine, leading to hypotension. The drug’s effect is 5 to 10 minutes after the start of the injection, and the peak effect is 15 to 30 minutes, with a half-life between one to two hours. According to the mentioned mechanism, the performance of the drug in improving the hemodynamic status in 60 and 90 minutes compared to 15 and 30 minutes from the beginning of surgery can be justified.

In a study presented in 2017 by Ahmadi et al. in Hamedan on 70 patients referred for rhinoplasty, patients were divided into one equal number of cases (receiving tranexamic acid) and control (receiving normal saline) in equal numbers. In general, they concluded that intravenous administration of tranexamic acid reduces bleeding, improves the surgeon's vision, and reduces the need for additional medication to reduce bleeding in rhinoplasty. Tranexamic acid is effective in controlling bleeding during surgery (22).

Frequency of complications during and after endoscopic sinus surgery and mean surgery time between the two groups receiving dexmedetomidine and tranexamic acid were not statistically significant. A notable point in both treatment groups was that none of the patients had major or life-threatening complications, and the severity of the minor side effects was such that there was no need for intervention to resolve them. One of the strong points of the study was blinding at the assessor level, and since the surgeon performed the intraoperative evaluation, the need for blinding at the patient level was not felt. Also, to prevent selection bias, in addition to blinding, blocks of 18 in a coated envelope were used so that the type of intervention for the next patient was not known. In order to prevent measurement bias, surgical procedures were performed by two expert surgeons. The limitation of the study was the small size of the sample, which could increase the chance of error. In order to control the selection bias and the confounding bias, in addition to the study design, the comparability of the two groups in terms of confounders was also controlled after the study and by statistical methods. It is recommended to conduct multi-centric studies with a larger sample size.

## Conclusion

In patients with chronic rhinosinusitis undergoing endoscopic surgery (with and without polyposis), to control bleeding during surgery, the use of injectable tranexamic acid has less bleeding volume and better quality of surgical field than dexmedetomidine. However, the effect of both drugs on the duration time of surgery and the number of complications are the same. Due to the better efficacy of tranexamic acid than dexmedetomidine in reducing the amount of bleeding during surgery, it is recommended to use tranexamic acid instead of dexmedetomidine in endoscopic surgery of chronic sinusitis (with and without polyposis).
